# Relationship between interprofessional collaboration and psychological distress experienced by healthcare professionals during COVID-19: a monocentric cross-sectional study

**DOI:** 10.3389/fmed.2024.1292608

**Published:** 2024-04-10

**Authors:** Kirstin Ruttmann, Sheila Albaladejo-Fuertes, Nicole Lindenberg, Claudia Kunst, Alexander Mehrl, Vera Kindl, Karsten Gülow, Sophie Schlosser-Hupf, Stephan Schmid, Martina Müller

**Affiliations:** ^1^Department of Internal Medicine I, Gastroenterology, Hepatology, Endocrinology, Rheumatology, and Infectious Diseases, University Hospital Regensburg, Regensburg, Germany; ^2^Nursing Development Department of the Care Management Head Office, University Hospital Regensburg, Regensburg, Germany; ^3^Department of Anaesthesiology, University Hospital of Regensburg, Regensburg, Germany

**Keywords:** interprofessional collaboration, interprofessional communication, interprofessional education, COVID-19 pandemic, mental well-being of healthcare professionals, mental health of health professionals, interprofessional collaborative practice, internal medicine

## Abstract

**Background:**

Since the onset of the COVID-19 pandemic, global healthcare systems have faced unprecedented challenges, leading to significant psychological distress among healthcare professionals. Recognizing the importance of enhanced interprofessional collaboration in alleviating this burden, as emphasized by the World Health Organization in 2020, we investigated whether such collaboration could mitigate staff psychological distress during crises. To our knowledge, no study has yet explored the role of interprofessional collaboration as a resilience factor in crises.

**Methods:**

For this monocentric cross-sectional study at a German university hospital, we examined the relationship between the quality of interprofessional collaboration and the psychological distress of healthcare professionals during the initial pandemic wave. We employed validated mental health instruments, such as the GAD-7 and PHQ-2, to assess anxiety and depressive symptoms. Additionally, custom-designed questionnaires evaluated “Pandemic-Associated Burden and Anxiety (PAB; PAA)” and interprofessional crisis management experiences. A novel “Interprofessional collaboration and communication (IPC)” assessment tool was developed based on international competency frameworks, demonstrating strong reliability.

**Results:**

The study involved 299 healthcare professionals (78.6% in direct contact with COVID-19 patients). Moderate levels of PAB/PAA were reported. However, a significant proportion experienced clinically relevant anxiety, as indicated by GAD-7. Negative IPC perceptions correlated with higher levels of psychological distress. Linear regression analysis showed associations between interprofessional collaboration and anxious and depressive symptoms, and pandemic-related burden.

**Conclusion:**

Our findings highlight the vital role of enhanced interprofessional collaboration in strengthening the psychological well-being of healthcare professionals during crises. The study underscores the need to foster a collaborative environment and integrate interprofessional education for resilience.

## 1 Introduction

The global COVID-19 pandemic has particularly emphasized the significance of interprofessional patient care (1), imposing considerable strain on healthcare systems and teams globally, initially reported from China (2, 3). Early data highlighted pandemic-related distress that could exacerbate mental health vulnerabilities for both the general population and healthcare professionals (4, 5). Especially during the early stages of the pandemic, mental symptoms such as anxiety and depression, along with pandemic-specific concerns, were evaluated (2). Specifically, staff directly involved in caring for COVID-19 patients experienced heightened psychological distress compared to their colleagues without direct exposure (6). While the psychological burden on healthcare professionals during the pandemic was generally found to be less severe than that experienced by the general population in most countries, there were clear international indications of increasing fatigue and dissatisfaction among these professionals approximately 2 years into the pandemic (6). Organizational key risk factors contributing to heightened psychological vulnerability included working in a university hospital, poor collaboration with colleagues, disruptions in daily routines (7), and a lack of organizational support (8).

In early 2020, the World Health Organization (WHO) issued recommendations urging leaders to mitigate psychological distress among healthcare professionals to enhance resilience (9). Particular emphasis was placed on strengthening interprofessional communication as a critical protective measure against psychological distress (10). Healthcare staff represent one of the most valuable resources during challenging crises, deserving protection. This includes not only safeguarding their physical health but also implementing approaches to promote mental well-being, thereby retaining resilient staff within clinical institutions and healthcare systems.

The call for enhanced interprofessional collaboration in healthcare predates the pandemic but has gained increased significance in its context of jointly addressing the crisis. However, prior to the COVID-19-related health crisis, only a small number of specific literature and empirical studies existed regarding the correlation between interprofessional collaboration and the manifestation of psychological distress in personnel during crises (11), including research related to coping with Ebola outbreaks (12). A limited number of pre-pandemic studies utilizing scoping reviews and qualitative analyses have shed light on interprofessional collaboration within mental health crisis response systems and intensive care unit dynamics during medical crises (13, 14). Furthermore existing literature suggested that deficient interprofessional collaboration correlated with lower job satisfaction, increased burnout prevalence, and higher job turnover rates, while strong collaboration, notably between physicians and nurses, appeared to be a potential protective factor against such distress (11, 15, 16). However, there are research gaps regarding the transferability of findings to large-scale societal crises like pandemics, larger study populations, psychometric control variables, and generalizability to both healthcare professionals and non-medical professionals in the healthcare sector.

Since 2019, the Department of Internal Medicine I at the University Teaching Hospital of Regensburg, Germany, has comprehensively integrated interprofessional collaboration into clinical practice through shared board meetings, case discussions, ward rounds, and joint teaching offerings. Additionally, an interprofessional training ward was established in the department to enhance team communication skills, which has been evaluated. At the onset of the pandemic, additional regular interprofessional briefings were initiated in each COVID-19 unit to ensure efficient information dissemination between managers and staff, along with pandemic-specific interprofessional teaching units.

The approach is grounded in the international definition of interprofessional collaboration within clinical settings, extending to educational environments where professionals from diverse disciplines come together to teach and learn collaboratively (17). This should foster a culture of interdisciplinary collaboration and knowledge exchange, crucial for ensuring seamless operations and facilitating smooth workflows through effective communication. Key elements that are essential for successful collaboration include open communication, smooth information flow, and patient-centered workflow coordination among various professional groups (17).

In summary, the COVID-19 pandemic has underscored an urgent need for effective measures to address psychological distress among healthcare personnel. Prioritizing the well-being of healthcare staff is essential for effectively managing daily clinical routines during a global pandemic. Despite the growing significance of interprofessional collaboration in healthcare, there remains a substantial research gap regarding its specific impact on the psychological health of staff during times of crisis. With the comprehensive interprofessional team approach established and the immediate implementation of WHO strategies to enhance extended interprofessional communication (see Supplementary Table 1), we had optimal access to evaluate these recommendations. To fill this gap, this study aims to explore whether there is a relationship between the quality of interprofessional collaboration and the onset of psychological distress among healthcare professionals during the initial months of the COVID-19 pandemic. Building upon this premise, our hypothesis suggests that intensified interprofessional collaboration and communication, perceived positively by those involved, may have served as protective measures against the onset of psychological distress among healthcare staff during this period. The present study aims to contribute to explaining potential correlations between interprofessional collaboration and crisis management and mental health. The findings of this research provide a basis for drawing conclusions regarding both proactive and crisis interventions within this context, as well as for future crises. The objective is to support interprofessional personnel and enhance their resilience.

## 2 Materials and methods

### 2.1 Background and objectives

The study, titled “Psychological Aspects of Interprofessionalism During the COVID-19 Pandemic” (PsyCoV-study), was conducted at the University Teaching Hospital of Regensburg in Germany. This hospital gained prominence for its role in treating acute COVID-19 patients during the initial phases of the pandemic. Specifically, the study examined the association between interprofessional collaboration, crisis management, and various mental health indicators, including pandemic-related burden, pandemic-associated anxiety, general anxiety levels, and depressive symptoms (see Supplementary Figure 1). The hospital administered a high volume of Extracorporeal Membrane Oxygenation (ECMO) therapies. In light of the extraordinary circumstances during the pandemic, characterized by the scarcity of comparable nationwide facilities capable of integrating both an interprofessional collaboration framework and a specialized intensive care center for COVID-19 patients, we made the strategic choice of adopting a monocentric approach.

### 2.2 Study design and sampling approach

Employing a monocentric, nonrandomized survey within a cross-sectional design, we aimed to investigate the relationship between interprofessional collaboration and the psychological distress experienced by healthcare staff during the initial wave of the pandemic. Additionally, our investigation carries an exploratory nature, as we seek to uncover potential novel insights and patterns within this domain. This approach enabled a comprehensive exploration of key factors influencing mental health indicators.

To ensure maximum coverage and representation within our study cohort, we conducted a census of all members of the interprofessional team during the survey period. We invited 775 employees to participate via paper-based questionnaires in order to maximize the representativeness of our sample and facilitate a thorough analysis of the study variables.

### 2.3 Data collection

Ethical clearance was obtained from the ethics committee of the University of Regensburg, ensuring adherence to European data protection standards. Data collection occurred between April 27, 2020, and May 12, 2020. Before the commencement of the survey, participants provided consent via paper-based forms. Strict anonymity was maintained throughout the survey process to protect participants’ privacy and confidentiality.

### 2.4 Participant inclusion criteria and classification approach

Included in the study were all employees from various professional groups within the defined areas who provided care for COVID-19 patients. Excluded were employees under the age of 18 years. The survey covered four general medical wards specializing in internal medicine, two intensive care wards specializing in internal medicine and anesthesiology, and functional areas such as the laboratory and endoscopy unit.

We engaged healthcare professionals, including physicians, nurses, physiotherapists, medical students who served as volunteer assistants for nurses following a specific training, and other allied professionals such as laboratory technologists, scientific staff, administrative personnel, and ward assistants who provided support in non-patient-facing activities. Distinctions were made between frontline healthcare workers, known to face elevated risks of infection and psychosocial stress, and second-line workers, who may experience lower infection risks but could still encounter stress due to organizational dynamics (2).

Furthermore, distinctions between medical and nonmedical staff (categorized as others with or without patient contact) within departments and interprofessional teams may yield insights into previously unexplored risk and stress factors for these groups. This nuanced approach facilitated a comprehensive assessment of the pandemic’s impact on interprofessional teams, potential protective aspects of interprofessional collaboration, and the formulation of targeted support measures for employees.

Due to data protection considerations, a more detailed classification based on participants’ departments, specific roles, or additional qualifications within the teams was not feasible. This requirement was imposed to prevent traceability and maintain participants’ anonymity.

### 2.5 Procedures

We employed several validated mental health instruments, including the German versions of the Generalized Anxiety Disorder Scale-7 (GAD-7) and the Patient Health Questionnaire-2 (PHQ-2), both of which are freely available for clinical and scientific purposes.

### 2.6 GAD-7, “Generalized Anxiety Disorder 7-Item Scale”

The GAD-7 covers the major diagnostic criteria for generalized anxiety disorder outlined in both DSM-IV and ICD-10. The German version of the GAD-7 was translated and validated by Löwe et al. (18) and exhibited robust internal consistency (Cronbach’s alpha = 0.89) and strong construct validity, as evidenced by its correlations with other anxiety scales. Scores on the GAD-7 range from 0 to 21, with a cutoff point of 15 indicating severe GAD. Items are rated on a 4-point Likert scale: (0) represents “not at all,” (1) “several days,” (2) “more than half the days,” and (3) “nearly every day.” For comparison with a norm sample from Germany, we referred to the reference value of *M* = 2.95, SD = 3.41 (18).

### 2.7 PHQ-2, “Patient Health Questionnaire-2”

To screen for depression, we utilized the Patient Health Questionnaire-2 (PHQ-2), an ultrashort version of the Patient Health Questionnaire-9 (PHQ-9), which focuses on the two main symptoms of major depression as outlined in DSM-IV (19). Similarly, the German version of the PHQ-2 was translated and validated by Löwe et al. (20) and displayed strong internal consistency (Cronbach’s Alpha = 0.83) and construct validity, showing significant correlations with other depression measures. The PHQ-2 score ranges from 0 to 6, with scores of 3 or higher likely indicating a major depressive disorder. For comparison with a norm sample from Germany, we used the reference value of *M* = 1.4, SD = 1.3 (20).

It should be noted that for both psychometric instruments, the GAD-7 and the PHQ-2, the comparisons with the German norm sample represent the general population before the pandemic. The selection of such a reference population facilitated a comprehensive evaluation of the mental health of our personnel at the onset of the crisis and enabled us to understand the implications of the situation.

### 2.8 Comprehensive assessment of the COVID-19 pandemic impact: “Pandemic-Associated Burden,” “Pandemic-Associated Anxiety,” and “Interprofessional Crisis Management”

In addition to employing standard measures, our questionnaire encompassed three distinct categorical sets of items: (1) “Pandemic-Associated Burden (PAB),” (2) “Pandemic-Associated Anxiety (PAA),” and (3) “Interprofessional Crisis Management (IPM)” experience. These categories were carefully selected to provide a comprehensive assessment of various facets of the pandemic’s impact.

Given the absence of pre-existing validated questionnaires tailored to these specific categories, customized questions were developed specifically for this study following an exploratory approach. Furthermore, owing to the acute crisis and regulations limiting the number of staff to be surveyed to minimize workload burdens, conducting a large-scale validation study was not feasible. Before data collection commenced, an expert panel conducted a face validation procedure to ensure the questionnaire’s clarity, address any potential errors or ambiguities, and confirm its relevance and appropriateness for the target audience. Subsequently, these questions underwent a meticulous review by a panel of hospital staff (*n* = 10). We ensured that the participants represented as wide a range as possible of professions, roles, and levels of experience. Adjustments to the wording of certain elements were made based on the feedback received during this pilot testing phase, aimed at improving the questionnaire’s clarity and effectiveness.

For “Pandemic-Associated Burden (PAB),” “Pandemic-Associated Anxiety (PAA),” and “Interprofessional Crisis Management (IPM),” we employed a sum score method to combine individual item scores into composite scores, enabling statistical analysis and interpretation of their respective performances. These scores are presented below their respective scales.

Three distinct categorical sets of items were included:

1. “Pandemic-Associated Burden (PAB)”: Participants were queried about “Pandemic-Associated Burden (PAB)” (see Supplementary Table 2). This section aimed to capture burdens at various levels, including organizational, societal, and personal. Items addressed concerns such as the flow of information in hospitals regarding the handling of the COVID-19 pandemic, the frustration of patients toward the healthcare system, and fear of infection. The self-assessment of PAB consisted of 16 items rated on a 6-point Likert scale, ranging from “low” (1) to “high” (6). The average cumulative score for PAB was 54, ranging from 16 to 96.
2. “Pandemic-Associated Anxiety (PAA)”: Participants were also queried about “Pandemic-Associated Anxiety (PAA)” (see Supplementary Table 2). Similar to PAB, this section aimed to assess anxieties related to the pandemic. Items addressed fears at organizational, societal, and personal levels. PAA consisted of 4 items rated on a 6-point Likert scale, ranging from “low” (1) to “high” (6). Both sets of categorical items were developed based on feedback from an interprofessional expert panel, with stressors described early in the pandemic by Lai et al. (2) serving as references. The average cumulative score for PAA was 14, ranging from 4 to 24.
3. “Interprofessional Crisis Management (IPM)” experience: The IPM section focused on experiences and perceptions of interprofessional collaboration during the crisis. Participants detailed their involvement in interprofessional support services such as briefings, team meetings, and specific training sessions (see Supplementary Table 3). This section consisted of 3 items regarding involvement and 2 items regarding perceptions, all rated on a 4-point Likert scale, ranging from “very poor” (4) to “very good” (1). The average cumulative score for IPM was 12.5, ranging from 5 to 20.

### 2.9 “Interprofessional Collaboration and Communication (IPC)”

Additionally, perceptions of the quality of interprofessional collaboration and communication during the pandemic were assessed. To facilitate measurement, we developed an assessment tool for interprofessional collaboration and communication, abbreviated as “IPC” (see Supplementary Table 4). To the best of our knowledge, no existing tool appropriately assesses interprofessional collaboration among experienced healthcare professionals providing care to adults in times of crisis. For operationalization, we followed international competency frameworks related to interprofessionality (21). We integrated characteristics such as “shared team goals,” “coordinated team approach,” “role clarity,” “equal communication,” “shared decision-making,” and “mutual support” (21). The IPC assessment consisted of 9 items, with an additional item addressing satisfaction levels. These were rated on a 4-point Likert scale: (4) “very poor,” (3) “rather poor,” (2) “rather good,” (1) “very good.” The average cumulative score for IPC was 22, ranging from 9 to 36.

Due to ethical considerations regarding employee protection during multiple surveys in an extraordinary crisis, a pre-test validation study for IPC could not be conducted. Therefore, we utilized the main study to assess and validate both the validity and reliability of the instrument. A Cronbach’s Alpha value of 0.925 indicates a high inter-item correlation within the IPC scale, demonstrating strong internal consistency. The high reliability, along with other indicators of construct validity (such as KMO, Bartlett’s Test, factor loadings, and explained total variance), suggests that our scale effectively measures the intended construct and provides reliable measurements.

Covariates included professional groups (physicians, nurses, ward assistants, medical students, physiotherapists, and others), gender, age group, marital status, weekly working hours, as well as contact with COVID-19 patients (clinical and non-clinical), and involvement in patient care tasks (direct or indirect). Gender information was not provided by two participants.

The assessment of psychological burdens across all tools was based on self-assessment, as was the evaluation of interprofessional collaboration and communication and interprofessional crisis management.

## 3 Statistical analysis

A power analysis was performed to estimate the necessary sample size. The significance was set at a level of *α* = 0.05, and a value of 0.95 was assumed for the statistical power. For t-tests included in the analysis, assuming a mean effect size of *d* = 0.5 and a not grossly unbalanced group distribution (*k* = 1), a necessary sample size of n1 = 88 and n2 = 88 (*n* = 176) was calculated. With regard to the *χ*^2^-tests, it was assumed that they would have a mean effect size of *w* = 0.3 with df = 5 degrees of freedom. This results in a necessary sample size of *n* = 220. The planned multiple linear regression analysis was estimated with *k* = 10 predictors and a mean effect size of *f* = 0.15. This results in a necessary sample size of n = 148. The ANOVA planned as part of the evaluation is estimated using k = 5 groups with a mean effect of *f* = 0.25. The resulting necessary sample size is *n* = 211. This results in a necessary sample size of *n* = 220 across all tests.

Descriptive statistics, including means, standard deviations, medians, and 95% confidence intervals, were calculated for demographic characteristics and variables, such as “Interprofessional Collaboration and Communication (IPC),” “Pandemic-Associated Anxiety (PAA),” “Pandemic-Associated Burden (PAB),” “Patient Health Questionnaire-2 (PHQ-2),” and “Generalized Anxiety Disorder-7 (GAD-7).” Pearson correlation analyses were conducted to examine associations between continuous variables. One-sample t-tests were employed to compare sample means with normative values. Linear regression analyses were performed to assess the relationship between interprofessional collaboration and mental symptoms, with GAD-7 and PHQ-2 serving as dependent variables, and dimensions of interprofessional collaboration as independent variables. Wilcoxon rank tests were utilized to evaluate differences in psychometric scales across subgroups, with gender, place of assignment, and profession considered as grouping factors. Chi-square tests were employed to explore associations between categorical variables and clinical abnormalities within subgroups. One-way ANOVA tests were utilized to investigate differences in IPC and “Interprofessional Crisis Management (IPM)” across various professional groups. Additionally, Kruskal-Wallis tests were conducted to accommodate non-normally distributed data where applicable. The significance level was set at *p* < 0.05 (two-tailed). Statistical analyses were performed using IBM® SPSS Versions 13.0 and 26.0.

## 4 Original results

A total of 299 healthcare professionals participated in the survey, yielding a response rate of 38.6%. Of these respondents, 22 were physicians (7.4%), 175 were registered nurses (58.5%), 43 were medical students (14.4%), 23 were other staff with direct contact with patients (7.7%), and 26 were other staff without contact with patients (8.7%). Out of the total, 235 (78.6%) were in direct contact with COVID-19 patients and were categorized as “frontline,” whereas 62 (20.7%) were not in direct contact, falling into the “second-line” category. The average weekly work hours were 35.6 (10.16) with a range of 8–80 h.

The average total scores for “Pandemic-Associated Anxiety (PAA)” and “Pandemic-Associated Burden (PAB)” were *M* = 3.07 (SD = 1.01) and *M* = 2.82 (SD = 1.03), respectively, with a maximum possible score of 6 points. These scores correspond to an intermediate level of distress. For the general cohort, the average sum score of the GAD-7, which assesses generalized anxiety, was *M* = 5.21, SD = 4.63, out of a possible 21 points. This translates to a low level of clinically relevant anxiety.

However, 139 participants (48.3%) had scores indicating a clinically relevant level of anxiety, falling at least in the “mild” range of 5 points in the total score of 21. The proportion of participants with a moderate likelihood of an anxiety disorder (using a GAD-7 cutoff of 10) was 17.4%. The average summative score for the PHQ-2, which evaluates depressive symptoms of major depression, was *M* = 1.49, SD = 1.43, out of a possible 6 points for the general cohort. Thus, the result of this summative score was not indicative of a clinically significant presence of depression.

However, in 60 cases (20.3%), the scores were at a clinically relevant level, falling within the range of 4–6 points. Established psychometric instruments, such as GAD-7 and PHQ-2, demonstrated significant correlations with our newly established questionnaires. Specifically, the GAD-7 showed a correlation with the “Pandemic-Associated Anxiety (PAA)” score (r = 0.531, *p* < 0.001), the “Pandemic-Associated Burden (PAB)” score (r = 0.679, *p* < 0.001), and depressive symptoms as measured by the PHQ-2 (*r* = 0.737, *p* < 0.001).

Compared to reference values from a norm sample of the German population, there was a significant difference in the GAD-7 scores across the entire sample, with a mean difference of 2.262 (t(287) = 8.284, *p* < 0.001). However, no significant difference was observed for PHQ-2 (t(294) = 1.174, *p* = 0.241). Notably, the group of nonmedical staff without patient contact showed the most pronounced differences, with a mean deviation (MD) of 3.746 (t (22) = 4.352, *p* < 0.001), while the nursing staff also exhibited substantial differences with an MD of 2.449 (t(167) = 6.409, *p* < 0.001). Both groups reported the highest levels of anxiety in our sample. Interestingly, medical students (MD = 1.515; t(42) = 2.290, *p* = 0.027) and nonmedical staff with patient contact (MD = 1.595; t(21) = 2.216, *p* = 0.038) also showed deviations from the norm, but these were more moderate. In contrast, physicians’ scores did not significantly deviate from the norm (MD = 1.459; t(21) = 1.589, *p* = 0.127), demonstrating they reported low levels of anxiety. For more detailed insights into the distribution of psychological burden across different demographic characteristics, refer to Table 1, which provides an analysis based on PHQ-2 and GAD-7 scores.

**Table 1:** Mean values and standard deviations of depressive and anxiety symptoms by demographic characteristics.

Mean values (M) and standard deviations (SD) regarding depressive and anxiety symptoms (GAD-7; PHQ-2) by demographic characteristics of the sample							
				PHQ-2		GAD-7	
		*N*	(%)	*M*	(SD)	*M*	(SD)
Male		87	29.1	1.221	(1.392)	4.276	(4.533)
Female		212	70.9	1.612	(1.444)	5.617	(4.629)
Age	<18	2	0.7	4.000	(2.828)	8.500	(4.950)
	18–25	67	22.9	1.478	(1.330)	5.448	(4.577)
	26–30	67	22.9	1.576	(1.436)	4.892	(4.610)
	31–40	67	22.9	1.652	(1.534)	5.923	(5.203)
	41–50	41	14.0	1.600	(1.516)	4.919	(4.310)
	>50	49	16.7	1.041	(1.274)	4.396	(4.211)
Single		123	42.1	1.620	(1.490)	5.393	(4.798)
In a relationship		169	57.9	1.407	(1.406)	5.050	(4.557)
Frontline		235	79.1	1.545	(1.431)	5.383	(4.729)
Second-line		62	20.9	1.274	(1.450)	4.373	(4.135)
Physicians		22	7.6	1.045	(1.253)	4.409	(4.306)
Medical students		43	14.9	1.023	(1.123)	4.465	(4.339)
Nurses		175	60.6	1.626	(1.511)	5.399	(4.953)
Others with contact		23	8.0	1.261	(0.964)	4.545	(3.377)
Others without contact		26	9.0	1.885	(1.705)	6.696	(4.128)

PHQ-2 (0–6), GAD-7 (0–21).

Within teams, the quality of communication with other professionals was most frequently rated as “good” (on a scale of 1–4; *M* = 2.02, SD = 0.77, 95% CI [1.93, 2.11]). The question regarding whether interprofessional collaboration improved during the pandemic was most often rated as “somewhat true” on a scale of 1–4; *M* = 2.42, SD = 0.99, 95% CI [2.29, 2.53].

**Figure 1:**
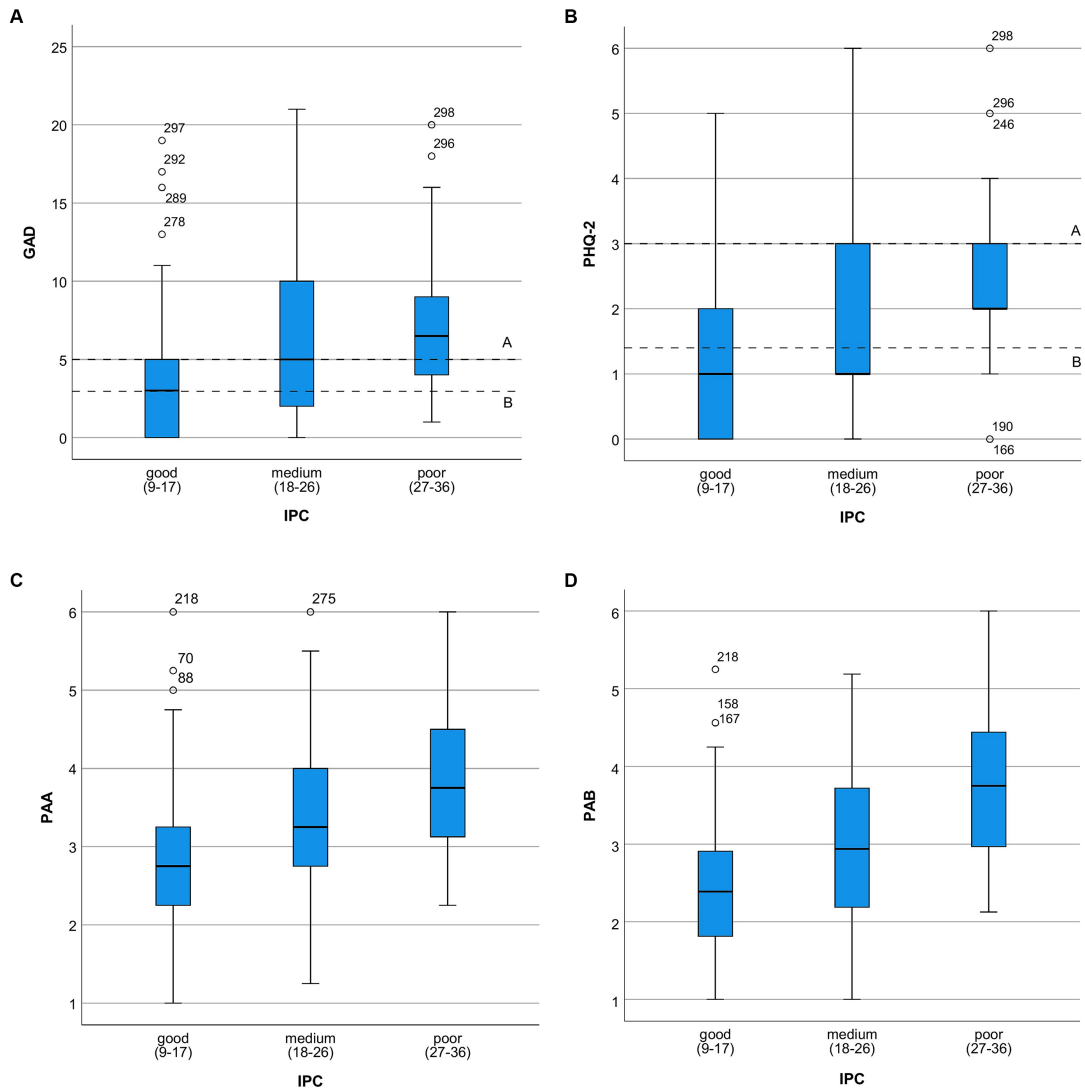
Degree of psychological burden in relation to the perceived quality of IPC. **(A)** Distribution of GAD-7^1^ scores by perceived quality of interprofessional collaboration and communication (IPC)^2^. [A = cut off ≥5; B = reference value of the normal population in Germany (before the pandemic)]; ^1^0–4, minimal anxiety; 5–9, mild anxiety; 10–14, moderate anxiety; 15–21, severe anxiety; ^2^ Sum score, 9–36; **(B)** Distribution of PHQ-2^1^ scores by perceived quality of interprofessional collaboration and communication (IPC)^2^ [A = cut off ≥3; B = reference value of the normal population in Germany (before the pandemic)]. ^1^PHQ-2 (0–6); 0–2 none; 3–6 hint for depression; ^2^ Sum score: 9–36; **(C)** Degree of middle Pandemic-Associated Anxiety (PAA)^1^ in relation to perceived quality of interprofessional collaboration and communication (IPC)^2^. The distribution of the frequencies of IPC^1^ with PAA; ^1^1–6 ascending; ^2^Sum score, 9–36; **(D)** Degree of middle Pandemic-Associated Burden (PAB)^1^ in relation to perceived quality of interprofessional collaboration and communication (IPC)^2^. ^1^1–6 ascending; ^2^Sum score, 9–36.

**Figure 2:**
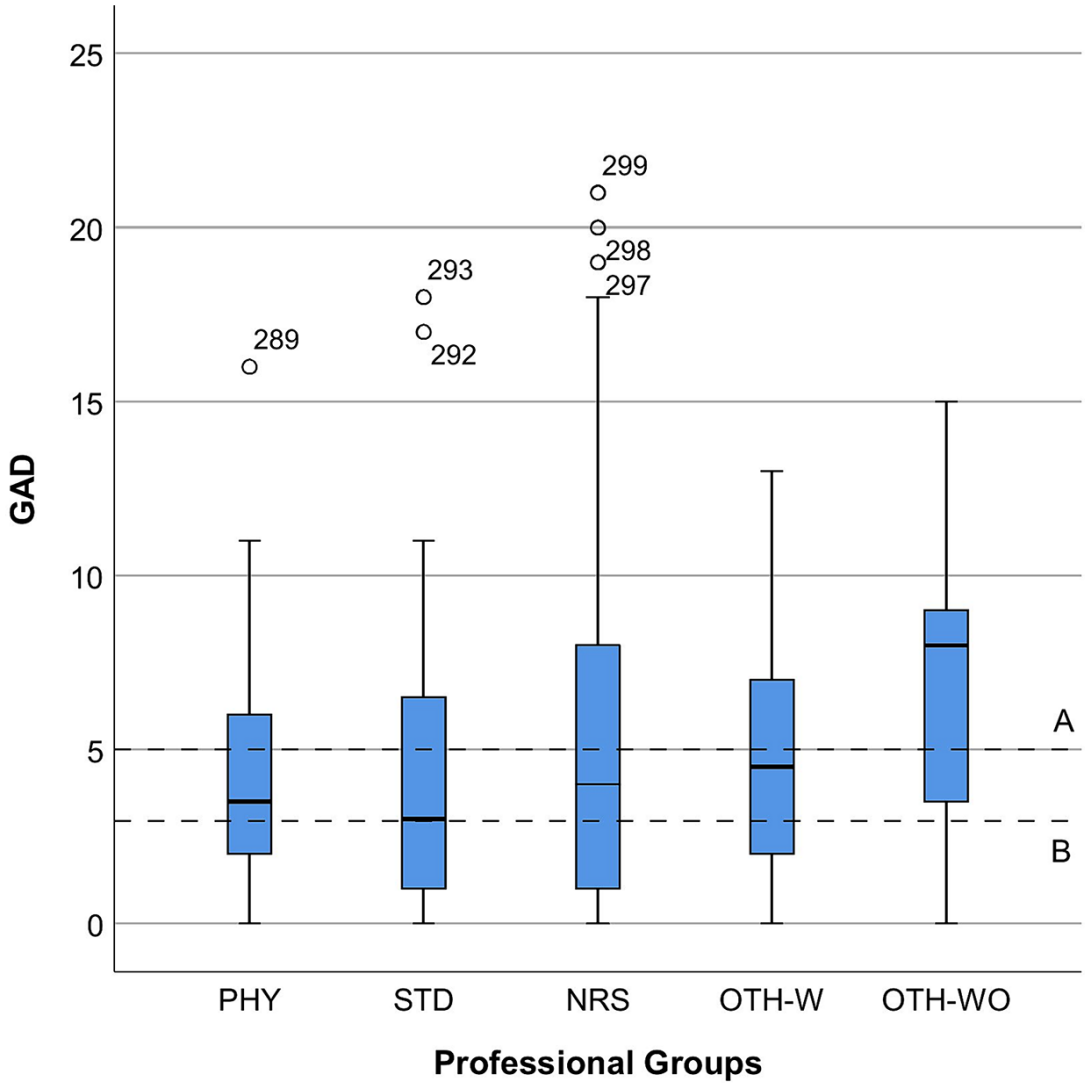
Distribution of GAD-7 scores by professional groups. PHY, physicians; STD, students; NRS, nurses; OTH-W, others with patient contact; OTH-WO, others without patient contact.

**Figure 3:**
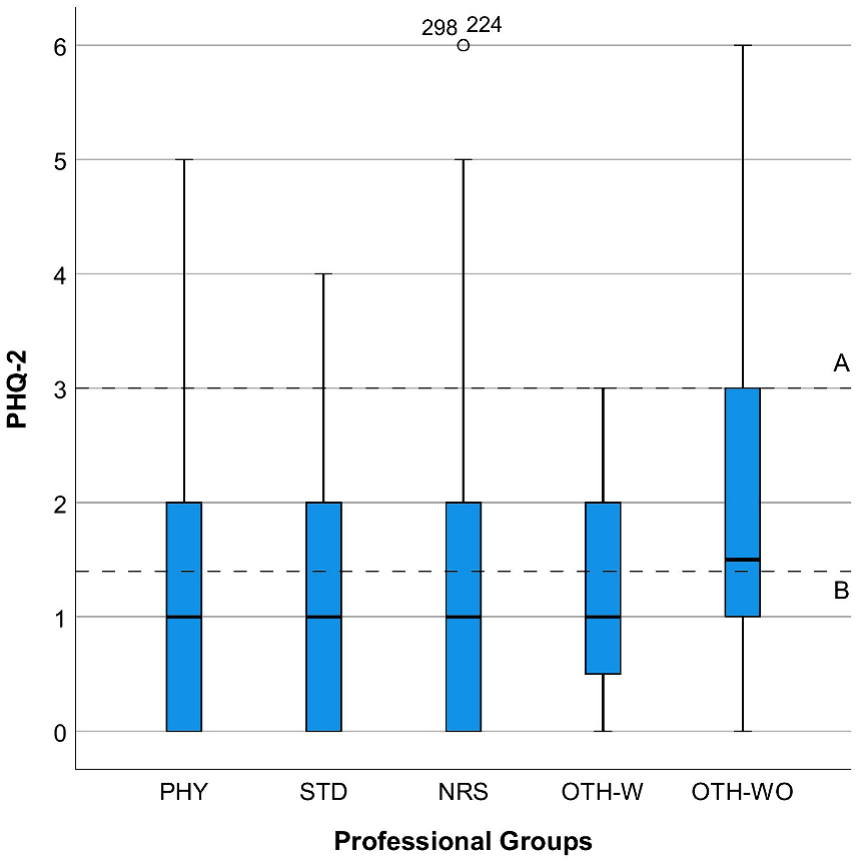
Distribution of PHQ-2 scores by professional groups. PHY, physicians; STD, students; NRS, nurses; OTH-W, others with patient contact; OTH-WO, others without patient contact.

However, for the “Interprofessional Collaboration and Communication (IPC)” scale, which ranged from 9 to 36, the mean score was 18.26 (SD = 5.82). This score corresponds to the experience of good team collaboration across various professional groups. Figure 1 shows the relationship between psychological distress and perceived quality of Interprofessional Collaboration and Communication (IPC). Subfigure (a) displays GAD-7 scores by IPC quality, while Subfigure (b) shows PHQ-2 scores. Subfigures (c, d) depict Pandemic-Associated Anxiety (PAA) and Pandemic-Associated Burden (PAB), respectively. Figure 2 illustrates GAD-7 scores by professional groups, providing insight into anxiety levels across different professions. Figure 3 presents PHQ-2 scores by professional groups, highlighting the prevalence of depression symptoms among various professions.

Regarding interprofessional briefings, 184 participants (62.8%) reported attending briefings within the past 4–6 weeks. Of these, 55 (18.8%) attended once, 19 (6.5%) weekly, and 47 (16.0%) daily. Both the flow of information within the organization and support for personal expertise were most frequently rated as “rather low.” Specifically, for a scale ranging from 1 to 4, the mean value was 3.43 (SD = 1.54, 95% CI [3.26, 3.61]) for the flow of information, and 3.40 (SD = 1.47, 95% CI [3.23, 3.57]) for support of personal expertise. This was despite the organization’s dedicated efforts to inform and support staff expertise. Importantly, negative evaluations of “Interprofessional Collaboration and Communication (IPC)” correlated with higher scores on the “Pandemic-Associated Burden” (PAB; *r* = 0.362, *p* < 0.001) and the “Pandemic-Associated Anxiety” (PAA; *r* = 0.335, *p* < 0.001). This suggests a relationship between perceived poor interprofessional collaboration and heightened psychological distress resulting from the pandemic.

As revealed by linear regression analysis, the assessment of “Interprofessional Collaboration and Communication (IPC)” was associated with the expression of both anxious symptoms (GAD-7) and depressive symptoms (PHQ-2) (R^2^ = 0.120, *F*(9,217) = 3.277, *p* = 0.001; R^2^ = 0.168, *F*(9,220) = 4.944, *p* < 0.001). Similarly, IPC was associated with the expression of “Pandemic-Associated Anxiety” (PAA; R^2^ = 0.148, *F*(9,222) = 4.286, *p* < 0.001) and “Pandemic-Associated Burden” (PAB; R^2^ = 0.167, F(9,222) = 4.945, *p* < 0.001). Anxiety symptoms as measured by the GAD-7 were most strongly influenced by the sub-items IPC-9, which represents “mutual support of team members” (*β* = −0.235, *p* = 0.014), and IPC-7, which stands for “open communication in the team” (*β* = 0.215, *p* = 0.023). There was also a trend towards an association with the sub-item IPC-5, representing “clarity about shared goals in the team” (*β* = 0.179, *p* = 0.052).

“Pandemic-Associated Anxiety (PAA)” and depressive symptoms as measured by PHQ-2 were most strongly associated with the item “open communication in the team” (IPC-7) (PAA; *β* = 0.200, *p* = 0.032 and PHQ-2; *β* = 0.167, *p* = 0.016). Similarly, assessments of “Interprofessional Crisis Management (IPM)” were associated with the expression of both anxious symptoms (GAD-7) and depressive symptoms (PHQ-2) (*R*^2^ = 0.108, *F*(5,180) = 4.370, *p* = 0.001; *R*^2^ = 0.097, *F*(5,184) = 3.941, *p* = 0.002).

Additionally, IPM assessments correlated with the expression of “Pandemic-Associated Anxiety” (*R*^2^ = 0.145, *F*(5,186) = 6.323, *p* < 0.001) and “Pandemic-Associated Burden” (*R*^2^ = 0.130, F(5,186) = 5.542, *p* < 0.001). Anxiety symptoms according to GAD-7 were most strongly associated with sub-items “preparation for patients with COVID-19 infection was well organized within the team” (IPM-3) (*β* = 0.240, *p* = 0.013) and “additional teaching opportunities and training opportunities” (IPM-2) (*β* = −0.204, *p* = 0.035). PAA was associated with sub-items “preparation for patients with COVID-19 infection was well organized within the team” (IPM-3) (*β* = 0.205, *p* = 0.029) and “team rounds” (IPM-1) (*β* = −0.187, *p* = 0.039), while depressive symptoms were associated with sub-item IPM-3 (*β* = 0.209, *p* = 0.031).

There was no evident impact of the information flow in the department on any of the psychological distress scores (GAD, PHQ-2, PAA, PAB) as indicated by the following F-statistics: *F*(5,284) = 1.292, *p* = 0.268; *F*(5,289) = 1.886, *p* = 0.097; *F*(5,292) = 1.458, *p* = 0.203; F(5,292) = 2.185, *p* = 0.056.

The frequency of interprofessional briefings demonstrated a significant protective effect against the expression of depressive symptoms (*F*(4,288) = 2.505, *p* = 0.042), indicating a statistical association rather than a causal relationship. However, no effect was observed on anxious symptoms as measured by GAD-7, “Pandemic-Associated Anxiety (PAA),” or “Pandemic-Associated Burden (PAB)” (*F*(4,282) = 1.593, *p* = 0.176; *F*(4,290) = 1.859, *p* = 0.118; *F*(4,292) = 1.700, *p* = 0.150). Training designed to enhance professional skills demonstrated a significant reduction in the expression of “Pandemic-Associated Anxiety (PAA)” (*F*(5,289) = 3.064, *p* = 0.010) and showed a trend towards reduced expression of depressive symptoms (*F*(5,286) = 2.237, *p* = 0.051). Table 2 offers insights into the correlation between IPC, mental symptoms, and the overall quality of interprofessional collaboration, and Table 3 shows the correlation between IPM strategies and psychological distress during the pandemic.

**Table 2:** Pearson correlation between interprofessional collaboration and communication items (IPC) with mental symptoms and the overall evaluation of interprofessional collaboration.

Pearson correlation between items of IPC communication and collaboration with mental symptoms (correlation coefficients)				
	PHQ-2	GAD-7	PAB	PAA
Communication with other professional groups	0.220**	0.184*	0.286**	0.188*
Decisions on patient care are made collaboratively	0.209**	0.160	0.265**	0.260**
Team members are working hand in hand	0.239**	0.233**	0.315**	0.271**
Different steps of care are well coordinated with each other	0.262**	0.202*	0.349**	0.277**
The goals of the interprofessional team are clear	0.219**	0.209**	0.277**	0.249**
Team members know their roles	0.314**	0.189*	0.305**	0.234**
Team members communicate openly with each other	0.313**	0.246**	0.356**	0.312**
Team members are assuming responsibilities	0.318**	0.202**	0.346**	0.297**
Team members help each other solve problems	0.187*	0.113	0.270**	0.208**
All in all, I have rated the interprofessional collaboration in my unit as inadequate	0.232**	0.189*	0.273**	0.238**

Pearson correlation between Items of IPC communication and collaboration with an evaluation of interprofessional collaboration in general (correlation coefficients)	
	Overall rating of interprofessional collaboration
Communication with other professional groups	0.701**
Decisions on patient care are made collaboratively	0.712**
Team members are working hand in hand	0.668**

This table displays the Pearson correlation coefficients between items assessing IPC and various mental symptoms, including PHQ-2 (depressive symptoms), GAD-7 (anxious symptoms), Pandemic-Associated Burden (PAB), and Pandemic-Associated Anxiety (PAA). Additionally, correlations between IPC items and the overall evaluation of interprofessional collaboration are presented. Significance levels: *p < 0.05; **p < 0.01; ***p < 0.001.

**Table 3:** Correlation between Items of Interprofessional Crisis Management (IPM) and mental symptoms.

	PHQ-2	GAD-7	PAB	PAA
Lack of additional team meetings to share information (COVID-19 pandemic)	−0.170*	−0.124	−0.191*	−0.249**
Lack of additional teaching and training activities (COVID-19 pandemic)	−0.216**	−0.240**	−0.265**	−0.207**
Preparation for patients with COVID-19 infection was poorly organized within the team	0.210**	0.201*	0.258**	0.301**
Interprofessional collaboration is poorer in the COVID-19 pandemic than before	0.163	0.119	0.184*	0.228**
Making use of the services (0/1)	−0.092	−0.058	−0.028	−0.046

This table presents the Pearson correlation coefficients between various items of IPM and mental symptoms, including depressive symptoms (PHQ-2), generalized anxiety symptoms (GAD-7), Pandemic-Associated Burden (PAB), and Pandemic-Associated Anxiety (PAA). The correlations were calculated to assess the relationship between crisis management strategies and psychological distress during the COVID-19 pandemic. Significance levels: *p < 0.05; **p < 0.01; ***p < 0.001.

Based on results from a Wilcoxon rank test, the female gender was identified as a significant risk factor for psychological distress in several measures (GAD-7: *Z* = −2.690, *p* = 0.007; PHQ-2: *Z* = −2.427, *p* = 0.015; PAB: *Z* = −2.819, *p* = 0.005), but not for PAA (*Z* = −0.762, *p* = 0.446). Frontline activities also emerged as significant risk factors for some measures of psychological distress (PAA: *Z* = −3.299, *p* = 0.001; PAB: *Z* = −3.529, *p* < 0.001) but not for GAD-7 (*Z* = −1.422, *p* = 0.155) or PHQ-2 (*Z* = −1.575, *p* = 0.115). For a breakdown of psychological distress by gender, consult Table 4 for detailed information. Table 5 provides detailed information on psychological distress, as well as perceptions of IPC and IPM, categorized by frontline and second-line roles.

**Table 4:** Gender differences in mental symptoms and Interprofessional Collaboration and Communication (IPC).

	Male		Female			
	*M*	(SD)	*M*	(SD)	*Z*	*p*
GAD-7	4.276	(4.533)	5.617	(4.629)	−2.690	0.007
PHQ-2	1.221	(1.392)	1.612	(1.444)	−2.427	0.015
PAA	3.033	(1.003)	3.095	(1.014)	−0.762	0.446
PAB	2.607	(1.053)	2.927	(1.011)	−2.819	0.005
IPC	18.579	(6.428)	18.115	(5.528)	−0.488	0.626
IPM	12.846	(2.539)	12.039	(2.904)	−1.644	0.100

This table displays the mean (M) and standard deviation (SD) for various mental symptoms and interprofessional collaboration scales, and Interprofessional Crisis Management (IPM) stratified by gender. Differences between genders were assessed using the Wilcoxon rank test, with reported Z-scores and corresponding *p*-values. GAD-7 General Anxiety Disorders (0–21); PHQ-2 Patient Health Questionnaire (0–6), PAA Pandemic-Associated Anxiety (1–6), PAB Pandemic-Associated Burden (1–6), IPC Interprofessional Collaboration and Communication (9–36), IPM Interprofessional Crisis Management (5–20).

**Table 5:** Disparities in mental symptoms and Interprofessional Collaboration and Communication (IPC) between frontline and second-line roles.

	Frontline		Second-line			
	*M*	(SD)	*M*	(SD)	*Z*	*p*
GAD-7	5.383	(4.729)	4.373	(4.135)	−1.422	0.155
PHQ-2	1.545	(1.431)	1.274	(1.450)	−1.575	0.115
PAA	3.177	(1.054)	2.698	(0.720)	−3.299	0.001
PAB	2.939	(1.061)	2.407	(0.788)	−3.529	0.000
IPC	18.226	(6.026)	18.355	(4.557)	−0.170	0.865
IPM	12.350	(2.721)	12.074	(3.339)	−0.495	0.621

The table displays the statistical analysis conducted using the Wilcoxon rank test to assess differences in psychometric scales and IPC between frontline and second-line roles. Mean values (M) and standard deviations (SD) are presented alongside Z-values and *p*-values for each scale. GAD-7 General Anxiety Disorders; PHQ Patient Health Questionnaire, PAA Pandemic-associated Anxiety, PAB Pandemic-Associated Burden, IPC Interprofessional Collaboration and Communication, IPM Interprofessional Crisis Management.

Furthermore, there were observed differences in psychological strain across occupational groups (GAD-7, χ^2^(4) = 5.792, *p* = 0.215; PHQ-2, χ^2^(4) = 9.162, *p* = 0.057; PAA, χ^2^(4) = 17.620, *p* = 0.001; PAB, χ^2^(4) = 14.772, *p* = 0.005). Table 6 contrasts the psychological distress and perceptions of IPC and IPM across various professional groups, offering insights into the disparities observed.

**Table 6:** Comparison of mental symptoms, Interprofessional Collaboration and Communication (IPC), and Interprofessional Crisis Management (IPM) across professional groups.

	PHY		STD		NRS		OTH-W		OTH-WO			
	*M*	(SD)	*M*	(SD)	*M*	(SD)	*M*	(SD)	*M*	(SD)	CHI^2^	*p*
GAD-7	4.409	(4.306)	4.465	(4.339)	5.399	(4.953)	4.545	(3.377)	6.696	(4.128)	5.792	0.215
PHQ-2	1.045	(1.253)	1.023	(1.123)	1.626	(1.511)	1.261	(0.964)	1.885	(1.705)	9.162	0.057
PAA	3.068	(0.897)	2.779	(0.858)	3.261	(1.072)	2.699	(0.760)	2.660	(0.907)	17.620	0.001
PAB	2.574	(0.916)	2.384	(0.779)	3.010	(1.082)	2.657	(1.029)	2.839	(1.002)	14.772	0.005
IPC	16.529	(5.363)	16.030	(3.687)	18.987	(6.186)	15.600	(4.695)	18.556	(6.307)	11.065	0.026
IPM	13.526	(2.836)	13.308	(2.323)	12.290	(2.797)	12.000	(2.867)	10.417	(2.275)	11.030	0.026

Statistical analysis was conducted using the Kruskal-Wallis test to compare mean scores across professional groups for each psychometric scale and scale of interprofessional collaboration, and Interprofessional Crisis Management (IPM). Mean (M) and standard deviation (SD) values are reported for each scale. Additionally, the Chi-square test was utilized to examine associations between professional groups and psychometric scales. GAD-7 General Anxiety Disorders; PHQ-2 Patient Health Questionnaire, PAA Pandemic-Associated Anxiety, PAB Pandemic-Associated Burden, IPC Interprofessional Collaboration and Communication, IPM Interprofessional Crisis Management; PHY physicians, STD students, NRS nurses, OTH-W others with patient contact, OTH-WO others without patient contact.

Regarding clinically relevant anxiety (GAD-7), significant differences were observed between genders (χ^2^(1) = 6.582, *p* = 0.010) and professional groups (χ^2^(4) = 9.768, *p* = 0.045), but not between age groups (χ^2^(5) = 4.548, *p* = 0.473) or activities corresponding to frontline or second-line (χ^2^(1) = 0.136, *p* = 0.771).

As shown by a one-way ANOVA, professional affiliation influenced the assessment of IPC and IPM in the total sample (IPC; *F*(4, 225) = 2.761, *p* = 0.029; IPM; *F*(4, 184) = 2.810, *p* = 0.027). However, *post hoc* tests revealed no significant difference between the professional groups. There was only a trend towards significance in the Bonferroni test between nursing (*M* = 18.987, SD = 6.186) and medical students (*M* = 16.030, SD = 3.687) with a mean difference of −2.957 (*p* = 0.081). This suggests that healthcare professionals with different professional affiliations may benefit from enhanced IPC.

## 5 Discussion

Our study aimed to comprehensively investigate the challenges faced by healthcare professionals during crises, particularly emphasizing the role of interprofessional collaboration and communication in hospital settings. Conducted during the initial wave of the COVID-19 pandemic in Germany, our study reveals a significant association between perceived interprofessional collaboration and mental symptoms, indicative of psychological distress, reported by healthcare professionals at a university teaching hospital.

### 5.1 Psychological distress observed in the cohort

Specifically, our study cohort primarily comprised frontline medical professionals directly involved in bedside care for COVID-19 patients, representing 78.6% of the participants. Although the cohort exhibited mild anxiety levels, with a mean GAD-7 score of 5.21, this represented a noticeable increase compared to anxiety levels in the general population before the pandemic (18). On the contrary, depressive symptoms, as assessed by the PHQ-2, were relatively low; however, 20.3% of respondents reported clinically relevant symptoms. Furthermore, additional analysis using specially designed questionnaires revealed moderate levels of “Pandemic-Associated Anxiety (PAA)” and “Pandemic-Associated Burden (PAB).” Both were positively correlated with anxiety symptoms, suggesting a connection between overall elevated anxiety and “Pandemic-Associated Burden and Anxiety (PAB; PAA).” General anxiety is often linked with fear, and it includes worries, avoidance, or unfounded fears. These results underscore the importance of a deeper understanding of the relationship between stress-induced anxiety and burden in an extraordinary crisis situation. Therefore, clinical institutions should cultivate sensitivity to recognize and be attentive to mental stressors and symptoms, enabling the provision of targeted interventions both proactively and in times of heightened stress.

### 5.2 Vulnerabilities and gender disparities

Of note, employees in nonmedical roles, such as administrative staff or ward assistants without direct patient contact, exhibited the highest levels of anxiety, as reflected in the GAD-7 scores, mirroring the anxiety levels found in the general population during the pandemic (22). This could potentially be attributed to a lack of pandemic-related knowledge and professional experience in addressing health challenges. The significant burden on collaborators without patient contact should remind leaders of the necessity for proactive crisis preparation and team-based crisis communication tailored to the recipients.

As described in previous studies, the exposure to patients with COVID-19 infection, particularly frontline activity, was confirmed as a significant risk factor for the development of pandemic-associated distress in our overall cohort (2, 23). In our study, nurses were found to be particularly vulnerable to psychological distress during the pandemic, especially those directly involved in frontline patient care. This heightened vulnerability may stem from the fact that nurses, who predominantly constitute this group, have the most frequent and closest contact with patients infected with COVID-19. It is important to note that women generally have a higher prevalence of depression and anxiety disorders within the population compared to men (24, 25).

In contrast, medical students working alongside nurses in our sample did not exhibit exceptional distress. This suggests that they likely benefited from structured interprofessional collaboration and a smooth transition to practice. Nevertheless, teams must pay special attention to volunteers within clinical routines (26). Across various medical professions, physicians demonstrated the highest level of resilience during the pandemic, likely due to their training and experience in handling high-pressure situations, resulting in the lowest degree of mental symptoms on average.

These findings highlight the importance of implementing tailored support strategies targeting specific demographic and occupational groups, including nonmedical staff. Gender- and workplace-sensitive approaches must be given attention, as they are deemed crucial aspects in promoting mental health, particularly in light of the heightened risk of negative psychological reactions among female frontline workers (2, 6, 23).

### 5.3 Role of interprofessional collaboration and team support in relation to psychological well-being

With our study, we were able to provide important insights that effective team communication and clearly defined goals have proven to be crucial factors in alleviating psychological stress among healthcare professionals. In contrast, inadequate communication and uncertainty regarding team goals have been identified as significant risk factors for heightened anxiety and stress-related burdens during the pandemic.

Encouragingly, collaborative decision-making processes and perceived team support have been associated with positive collaborative experiences (17, 27, 28). Additionally, we have identified supplementary team factors that influence team management. Individuals who have felt that their teams were ill-prepared for patients with COVID-19 infections have been more likely to report depressive symptoms and anxiety. This higher anxiety was associated with negative perceptions of “Interprofessional Collaboration and Communication (IPC).” In this context, it can be inferred that elevated anxiety might detrimentally impact both the perception of interprofessional teamwork and the capability to collaborate. On the other hand, lower anxiety and depression levels might enable individuals to work more effectively and efficiently within a team (29). Our analysis demonstrates that negative evaluations of interprofessional collaboration/communication show a moderate yet noteworthy correlation with both “Pandemic-Associated Burden” (*r* = 0.362, *p* < 0.001) and “Pandemic-Associated Anxiety” (*r* = 0.335, *p* < 0.001). In a practical context, these findings imply that if professionals across various disciplines view collaboration and communication negatively, they are more likely to encounter heightened levels of stress and anxiety associated with the pandemic. Despite the moderate correlation strength, the potential influence of fostering interprofessional collaboration and communication on the psychological well-being of healthcare staff is evident.

Our findings highlight the critical importance of interprofessional communication and team support for promoting employee mental well-being during periods of crisis (17). Effective team communication and clearly defined goals were identified as pivotal factors in alleviating psychological distress among healthcare professionals. Conversely, inadequate communication and ambiguity regarding team objectives emerged as significant risk factors for heightened overall anxiety and “Pandemic-Associated Anxiety (PAA).”

### 5.4 Role of interprofessional crisis management in relation to psychological well-being

Furthermore, our study underscores the effectiveness of “Interprofessional Crisis Management (IPM)” interventions, such as interprofessional briefings and training sessions, in reducing anxiety and depressive symptoms among staff. These findings emphasize the importance of proactive crisis preparation and ongoing professional development initiatives in fostering resilience and mitigating pandemic-induced stress.

In our survey, over half of the staff reported participating in interprofessional briefings, with 22.5% doing so daily or at least once a week. Training aimed at enhancing professional skills significantly impacted the severity of anxiety in the context of the pandemic, highlighting its crucial role in interdisciplinary crisis management at the organizational level. Regarding effective crisis management, factors such as collaborative management and clarification of roles and responsibilities within teams have previously been identified as essential strategies in high-pressure environments (12, 27, 30). Correspondingly, inadequate professional support during the COVID-19 crisis was linked to increased psychological distress in at least two studies (27, 30). These factors were corroborated in our study.

### 5.5 Summary of the central findings of our study

In conclusion, our study underscores the value of robust interprofessional collaboration, emphasizing open communication, defined team goals, and shared decision-making in enhancing resilience among healthcare professionals during challenging times (17). Our study not only highlights the value of strong interprofessional collaboration and communication but also underscores the paramount importance of fostering rigorous interprofessional approaches to strengthen the well-being of healthcare staff amidst crises (1). Notably, a suboptimal perception of Interprofessional Crisis Management (IPM) emerged as a risk factor for depressive symptoms and inadequate team communication correlated with heightened risks of anxiety, depressive symptoms, and increased pandemic-induced stress.

With our study, we addressed a gap in the literature. When examining the literature to contextualize our findings within the work of other authors, there are indications of the effects of interprofessional collaboration on staff outside of pandemics and crises. For instance, Vermeir et al. (31) found a positive correlation between satisfaction with communication and nurses’ job satisfaction. This manifested in reduced turnover intentions and a decreased risk of burnout. Subsequently, Labrague et al. (32) described interprofessional collaboration as a mediator between nurses’ work environment and job satisfaction. Our findings support these assertions, insofar as positively rated interprofessionalism can contribute to increased well-being for the staff, particularly for the nursing group. Amidst a major crisis such as the COVID-19 pandemic, some research has identified interprofessional collaboration or teamwork as apotential protective factor among others, influencing the mental strain experienced by healthcare professionals. However, the relationships and individual impacts have not yet been investigated as a standalone research question concerning crisis management and mental well-being (33, 34).

### 5.6 Implications and recommendations for practice in the post-pandemic era

Based on our findings, we strongly advocate for healthcare leaders to prioritize interprofessional initiatives aimed at supporting staff well-being and optimizing patient care. Implementing tools such as interprofessional briefings and education programs can serve as invaluable resources for promoting resilience and enhancing competent crisis management in healthcare settings. Looking ahead to future crises, proactive initiatives are needed to address these challenges. One such initiative could entail the establishment of interdisciplinary task forces comprising professionals from diverse healthcare disciplines. These task forces, akin to rapid response teams, would collaborate to devise comprehensive strategies for stress management, communication enhancement, and teamwork promotion across various departments. By bringing together experts from different fields, such task forces can facilitate the swift implementation of evidence-based practices and innovative solutions to emerging challenges. Regular interprofessional huddles or debriefing sessions, led by task force members, serve as effective means to enhance real-time communication and problem-solving among team members, thus promoting a cohesive approach to patient care.

As a relatively quick and low-threshold program, the University Teaching Hospital of Regensburg is implementing stress managers post-pandemic. These stress managers have undergone a certified training program. They are embedded within the teams to provide on-site counseling, interventions, and training for interprofessional staff, all aimed at stress prevention. Furthermore, investment in cross-disciplinary training programs focused on honing interprofessional collaboration skills, conflict resolution techniques, and shared decision-making abilities can equip staff with the requisite tools and competencies to function effectively as a cohesive team.

By fostering a culture grounded in mutual respect, trust, and collaboration, healthcare institutions can harness the collective expertise of multidisciplinary teams to tackle intricate challenges and optimize patient outcomes (31). Embedding interprofessional collaboration principles into organizational policies, protocols, and performance evaluations further underscores the significance of teamwork and collaboration in attaining shared objectives. Ultimately, prioritizing rigorous interprofessional approaches empowers healthcare institutions to cultivate resilient, cohesive teams proficient at navigating crises with balance and empathy. Such approaches must be integrated into healthcare curricula and teaching methodologies, ensuring that future healthcare professionals are equipped with the necessary skills for effective collaboration and crisis management (35).

### 5.7 Limitations of the study and need for future research

While our study has provided valuable insights, it is important to acknowledge several limitations. First, there is the issue of selection bias due to voluntary participation, which may have led to sample distortion as individuals who chose to participate may possess different characteristics or opinions compared to those who did not. Extending our findings to other hospital settings requires consideration, as various contextual factors such as the hospital’s organizational structure, geographical location, and specific operational dynamics may influence outcomes differently. Additionally, the underrepresentation of physicians in our sample may limit the generalizability of our findings and introduce bias in interpretation, as their perspectives and experiences may not have been adequately accounted for. Despite these limitations, our decision to conduct a direct comparative analysis between physicians (*n* = 22) and nurses (*n* = 175) was based on the relevance of both groups to our research interests and their potential influence on the variables under investigation. Physicians and nurses operate in similar clinical environments and often share overlapping tasks directly related to the variables under study. The results of this comparison can provide crucial insights that are relevant for both theoretical advancement and practical application, particularly in clinical settings where these two professional groups collaborate closely and complement each other.

Furthermore, the absence of pre-existing anxiety level data among participants before the COVID-19 pandemic complicates the interpretation of our results, as other factors outside the pandemic context may have influenced the measured anxiety levels. For an external assessment of the burden of psychological symptoms in our study population using the PHQ-2 and GAD-7, we opted for a reference population consisting of a norm sample from the German population before the pandemic. During the initial wave of the pandemic, while much of the world implemented lockdown measures and many hospital workers transitioned to remote work, our sample of healthcare professionals, including allied health practitioners, continued to operate in clinical settings without clear medical guidelines, vaccines, or established treatment options. Due to the high prevalence of ECMO therapies and the associated significant burden, as well as the numerical imbalance in professional subgroups (such as a higher number of nurses compared to physicians), direct comparisons with other healthcare professionals from studies are prone to bias and challenging to interpret. The utilization of a norm sample from the pre-pandemic German population enabled us to contextualize our findings and assess the psychological impact on our cohort.

Moreover, our study was monocentric and not a randomized controlled trial, which could impact the generalizability and causal inferences of our findings. We also lacked information on participants’ specialty areas or potentially more stressful activities, such as ECMO therapy, although we differentiated between frontline and second-line roles.

Furthermore, our study did not compare the “Interprofessional Collaboration and Communication (IPC)” instrument, developed specifically for this study, with another assessment tool, potentially impacting the validity of our measure. While such a comparison is not obligatory to establish the validity of our instrument, it could strengthen its reliability and robustness. Similarly, the same assumption applies to the self-designed questionnaires concerning “Pandemic-Associated Burden (PAB),” “Pandemic-Associated Anxiety (PAA),” and “Interprofessional Crisis Management (IPM),” which are grounded in literature-based evidence and the guidance of an expert panel.

Our investigation reveals moderate correlations between the quality of “Interprofessional Collaboration and Communication (IPC)” and the scores for “Generalized Anxiety Disorder-7 (GAD-7),” “Pandemic-Associated Anxiety (PAA), and Burden (PAB)” (all *p* < 0.05) in our sample. These findings suggest that improved IPC is associated with a reduced degree of psychological distress during the pandemic. This relationship is moderate most likely due to the overall moderate levels of psychological distress and the influences of the sample sizes with respect to the different subgroups of healthcare workers. This connection sheds light on the complex interactions between IPC and psychological impacts during the pandemic. While strong correlations indicate robust associations, moderate and weak correlations provide valuable insights into these intricate relationships, highlighting the need for further investigation and targeted interventions to support healthcare professionals during times of crisis. In the overall scope of the study results, it is also important to note that the cross-sectional nature of our study limits our ability to infer long-term effects.

Moving forward, it is imperative for future research to explore the comparative validity of IPC with established instruments, conduct multicentric studies with randomized controlled designs, and collect comprehensive mental health data. This includes measurements of anxiety levels, depression levels, and other relevant psychological factors. To confirm our results, it is crucial that future studies examine and further elucidate the complex interplay between the quality of interprofessional collaboration and psychological well-being, quantified using validated psychometric tests.

## 6 Conclusion

Our study, with its focus on interprofessionalism, underscores the necessity for healthcare institutions to sensitively address the needs and requirements of all team members, whether medical or nonmedical staff. This is crucial because during daily routines and especially amidst periods of high workload and crises, all members contribute to overcoming challenges and ultimately delivering outstanding patient care. Such approaches could significantly contribute to strengthening the resilience of interprofessional healthcare teams and mitigating turnover rates attributable to psychological distress. By prioritizing rigorous research and evidence-based interventions aimed at improving mental health outcomes, healthcare organizations can create supportive environments that enhance job satisfaction and overall well-being. Thus, investing in strategies to support the psychological strength and adaptability of interprofessional teams is essential for fostering a sustainable and thriving healthcare workforce.

## Data availability statement

The datasets presented in this article are not readily available due to privacy considerations for the surveyed employees. Requests to access the datasets should be directed to Kirstin.Ruttmann@ukr.de.

## Ethics statement

In this study, strict adherence to applicable data protection policies has been ensured to safeguard participant confidentiality. Particular attention has been paid to ensuring non-attribution to individual participants. These measures are crucial to maintaining anonymity and upholding ethical standards.

## Author contributions

KR: Conceptualization, Data curation, Formal analysis, Investigation, Methodology, Project administration, Validation, Visualization, Writing – original draft. SA-F: Conceptualization, Investigation, Methodology, Project administration, Writing – review & editing. NL: Data curation, Formal analysis, Validation, Writing – review & editing. CK: Data curation, Supervision, Validation, Writing – review & editing. AM: Investigation, Writing – review & editing. VK: Writing – review & editing. KG: Conceptualization, Project administration, Supervision, Writing – review & editing. SS-H: Writing – review & editing. SS: Investigation, Supervision, Writing – review & editing. MM: Conceptualization, Data curation, Investigation, Methodology, Project administration, Supervision, Validation, Writing – review & editing.
