# Supplementary material for: Relationship between interprofessional collaboration and psychological distress experienced by healthcare professionals during COVID-19: a monocentric cross-sectional study
Source: Front Med (Lausanne). 2024 Apr 10;11:1292608. doi: 10.3389/fmed.2024.1292608 (PMC11039835; doi:10.3389/fmed.2024.1292608)
Supplement: Supplementary file 1 [file Data_Sheet_1.docx]

Supplementary Material

**Table 1** Overview of activities of strengthened interprofessional communication and collaboration and organizational support in the hospital

**Table 2** Pandemic – Associated Burden/Anxiety (PAB/PAA) questionnaires

**Table 3** Interprofessional Crisis Management (IPM) questionnaire

**Table 4** Interprofessional Communication and Collaboration (IPC) questionnaire

**Figure 1** Synopsis of the study design

| **Activities at the department/ward level (intensified and pandemic-focused)** | **Services at the organizational level**  **(intensified and pandemic-focused)** |
| --- | --- |
| **Primary strategy of defined interprofessional collaboration and communication (since 2019)** |  |
| - Regular shared board meetings of all relevant professional groups and disciplines (with and without patient contact) | - Communication and information meetings for all employees |
| - Daily ward rounds to coordinate activities (crisis management) | - Centralized training programs on hygiene and prevention of transmission of infections, educational sessions, and general aspects of pandemic management |
| - Daily Morning briefings to coordinate activities and give pieces of information to the staff (crisis management) of all relevant professional groups and disciplines (with and without patient contact) | - Open-access e-learning and e-information resources |
| - Regular interprofessional education and facilitation | - Special training sessions and facilitation for nurses with pandemic-related rotations in the wards |
| - A fundamental strategy of an interprofessional research collaboration of all relevant professional groups and disciplines (with and without patient contact) | - Special training sessions and facilitation for medical students as volunteers in the wards |
|  | - Contact persons in important and central departments for pandemic management (infectiology, hygiene, education center, quality management, occupational medicine, nursing management, etc.) |
|  | - Newsletters and recommendations for managers and employees (online/paper-based) |
|  | - Clinical and operational partnerships between leaders to ensure a safe environment for patients and staff |
|  |  |
|  | - Provision of a telephone hotline for mental health problems and counseling services for all employees by a team of psychologists |

**Supplementary Table 1.** Overview of activities of strengthened interprofessional communication and collaboration and organizational support in the hospital

|  | |
| --- | --- |
| **Number Items included in the questionnaire** | |
| PAB (01) | Fear of death |
| PAB (02) | Concerns to be socially isolated |
| PAB (01) | Feeling of being helpless |
| PAB (02) | Concern about separation from one's own community/social contacts |
| PAB (03) | Experiences from previous health crises |
| PAB (04) | Stigmatization due to contact with infectious patients |
| PAB (05) | Strict safety measures |
| PAB (06) | Permanent need for concentration |
| PAB (07) | Increased workload/ high pressure for further training |
| PAB (08) | Decreased social support |
| PAB (09) | Reduced self-care due to stress/strain |
| PAB (10) | Insufficient information about long-term exposure to COVID-19-positive patients |
| PAB (11) | Concerns about the exposure of own family/relatives |
| PAB (12) | Frustration of patients/families on health system |
| PAB (13) | Feeling isolated from the team due to care of infectious patients |
| PAB (14) | Worries about the increased workload of colleagues in case of own illness |
| PAA (15) | Perceived fear among colleagues |
| PAA (16) | Individual fear of the pandemic |
| PAA (17) | Fear of (own) severe infection (COVID-19) |
| PAA (18) | Individual risk of severe infection (COVID-19) |

**Supplementary Table 2.** Pandemic – Associated Burden/Anxieties (PAB/PAA) questionnaire

|  | |
| --- | --- |
| **Number Items included in the questionnaire** | |
| IPM (01) | Supplemental team roundtables were held to share information (COVID-19 pandemic) |
| IPM (02) | Supplemental teaching and training opportunities were available (COVID-19 pandemic) |
| IPM (03) | Preparation for patients with COVID-19 infection was well organized within the team |
| IPM (04) | Interprofessional collaboration improved during the COVID-19 pandemic |
| IPM (05) | Use of additional services |

**Supplementary Table 3. Interprofessional Crisis Management (IPM) questionnaire**

|  | |
| --- | --- |
| **Number Items included in the questionnaire** | |
| IPC (01) | Communication with other professional groups |
| IPC (02) | Decisions on patient care are made collaboratively |
| IPC (03) | Team members are working hand in hand |
| IPC (04) | Different steps of care are well coordinated with each other |
| IPC (05) | The goals of the interprofessional team are clear |
| IPC (06) | Team members know their roles |
| IPC (07) | Team members communicate openly with each other |
| IPC (08) | Team members are assuming responsibilities |
| IPC (09) | Team members help each other solve problems |

**Supplementary Table 4.** Interprofessional Communication(Collaboration) (IPC) questionnaire

**Inclusion criteria**

Health and allied health professionals with and without patient contact in departments/wards treating patients with acute COVID-19 infection.

**Pandemic-Associated Burden/Anxiety**

PAA/PAB

Covariates: Professional groups (physicians, nurses, ward assistants, medical students, physiotherapists, and others), gender, age group, marital status, and working hours.

**Intervention**

Intensified interprofessional communication and pandemic-related crisis management (at the departmental and ward levels) and additional support services provided by the organization.

**GAD-7**

**PHQ-2**

**Survey period and data collection from 04/27/20 to 05/12/20** (1st Covid-19 wave in Germany)

**Interprofessionalism**

Interprofessional communication and collaboration"

**Crisis management: “Covid-19 pandemic“**

Support by the organization /crisis management

**Supplementary Figure 1.** Synopsis of the Study design “Study on psychological aspects of staff in the context of interprofessionalism during the CoV-19 pandemic” (PsyCoV-study)

**
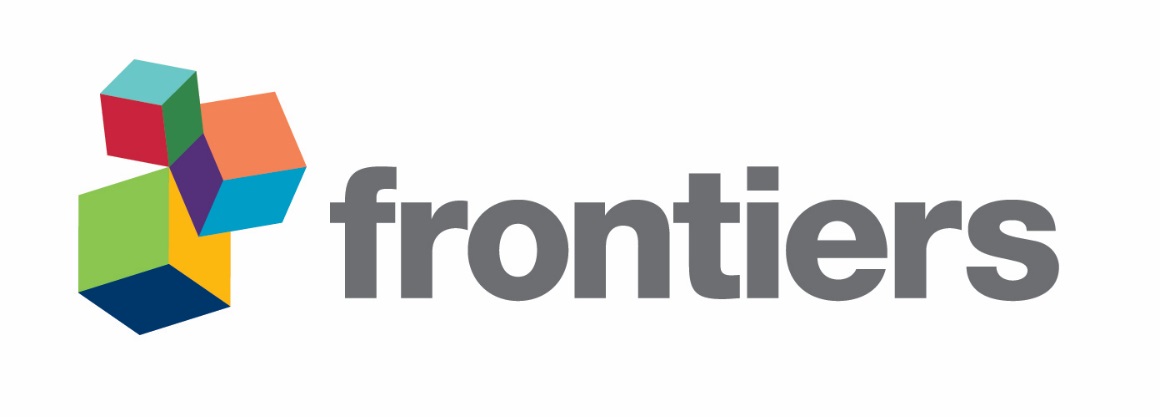
**
